# Circovirus in Blood of a Febrile Horse with Hepatitis

**DOI:** 10.3390/v13050944

**Published:** 2021-05-20

**Authors:** Alvin Hui, Eda Altan, Nathan Slovis, Caitlin Fletcher, Xutao Deng, Eric Delwart

**Affiliations:** 1 Vitalant Research Institute, San Francisco, CA 94118, USA; ahui@vitalant.org (A.H.); edaltan@hotmail.com (E.A.); xdeng@vitalant.org (X.D.); 2 Department of Laboratory Medicine, University of California at San Francisco, San Francisco, CA 94118, USA; 3 Hagyard Equine Medical Institute, 4250 Iron Works Pike, Lexington, KY 40361, USA; nslovis@hagyard.com (N.S.); cfletcher@hagyard.com (C.F.)

**Keywords:** *Equues caballus*, *Circoviridae*, viral metagenomics, hepatitis

## Abstract

Circoviruses infect vertebrates where they can result in a wide range of disease signs or in asymptomatic infections. Using viral metagenomics we analyzed a pool of five sera from four healthy and one sick horse. Sequences from parvovirus-H, equus anellovirus, and distantly related to mammalian circoviruses were recognized. PCR identified the circovirus reads as originating from a pregnant mare with fever and hepatitis. That horse’s serum was also positive by real time PCR for equine parvovirus H and negative for the flavivirus equine hepacivirus. The complete circular genome of equine circovirus 1 strain Charaf (EqCV1-Charaf) was completed using PCR and Sanger sequencing. EqCV1 replicase showed 73–74% identity to those of their closest relatives, pig circoviruses 1/2, and elk circovirus. The closest capsid proteins were from the same ungulate circoviruses with 62–63% identity. The overall nucleotide identity of 72% to its closest relative indicates that EqCV1 is a new species in the *Circovirus* genus, the first reported in genus Equus. Whether EqCV1 alone or in co-infections can result in disease and its prevalence in different equine populations will require further studies now facilitated using EqCV1′s genome sequence.

## 1. Introduction

Circoviruses are small viruses in the *Circoviridae* family whose circular single-stranded DNA genomes of ~2 kb are amongst the smallest known [1]. Circoviruses are associated with a wide spectrum of disease ranging from asymptomatic to lethal in different birds, pigs, elks, cows, dogs, panda bears, bears, bobcats, pumas, foxes, minks, palm civets, fur seals, numerous rodents, as well as in reptiles and fish [2,3,4,5,6,7,8,9,10,11,12,13,14,15,16,17,18,19,20]. Circoviruses have also been described in numerous bat species [21,22,23,24,25,26,27,28]. 

Here we report on a new circovirus we called equine circovirus 1 strain Charaf (EqCV1-Charaf) identified during a viral metagenomics analysis of plasma from horses.

## 2. Materials and Methods

Viral-like particles were enriched by filtration and digestion with nucleases enzymes to reduce the concentration of non-capsid protected nucleic acids. Following nucleic acids extraction and random RT-PCR the DNA amplification products were converted to Illumina compatible DNA using Nextera™ XT Sample Preparation Kit with dual barcoding [29] and analyzed on a Illumina MiSeq using 250 bases paired end sequencing. These reads were submitted to GenBank under Biosample accession number SAMN18737161. Bioinformatics analyses were conducted as described using BLASTx to detect reads and contigs showing translated protein sequences similarity to all know eukaryotic viruses in RefSeq of GenBank [30].

A pair of PCR primers was designed based on circovirus-related reads to generate a 352 bp amplicon (CircoF: 5′TGTCGAAGCTCTCTTCAGGAG3′ and CircoR: 5′ATGTGGCTGAACCTAGACACCC3′ using 40 cycles of 95 °C melt 30 s, 57 °C anneal 30 s, 72 °C extension 90 s) and used to test nucleic acids extracted individually from each of the five sera in the pool. 

The DNA genome of the circovirus was then completed using two PCR over gaps with primers designed from the short reads (available on request) and the amplicons directly Sanger sequenced by primer walking. Sequence analysis was performed using MAFFT multiple sequence alignment [31]. Sequence identity matrix was measured using BioEdit. To identify the stem-loop structure, the nucleotide region upstream of the main ORFs was analyzed with Mfold [32]. Phylogenetic trees with 100 bootstrap resamples of the alignment data sets were generated using the maximum likelihood method and visualized using the program MEGA version X [33]. Bootstrap values (based on 100 replicates) for each node are shown if >70%.

## 3. Results

Sera from five horses from Kentucky were pooled and analyzed by viral metagenomics. Four horses were clinically healthy while one had fever and hepatitis. Analysis of the resulting 120,000 Illumina sequence reads showed the presence of fifteen reads to anellovirus Torque teno equus virus 1, two reads to the recently described equine parvovirus H and forty-five reads with best hits to different circoviruses. Because anelloviruses are considered commensal viruses and the genome and pathogenicity of equine parvovirus H are well characterized we focused on the circovirus hit with BLASTx E score ranging from 0.003 to 10^−27^ indicating the detection of a “novel” circovirus. 

Each plasma sample was individually extracted and tested by PCR for the presence of circovirus DNA. Only one serum collected from a 10 months pregnant mare stabled in central Kentucky was PCR positive. The circovirus infected horse exhibited pyrexia and inappetence with elevated liver enzymes GGT (Gamma Glutamyl Transferase) and SDH (Sorbitol dehydrogenase) activities. The horse responded favorably to NSAIDS with Flunixin PRN for fevers, antibiotics TMS tablets (Trimethoprim 160 mg and Sulfamethoxazole 800 mg) 24 mg/kg BID, Pentoxifylline 4 g BID, Vitamin E (NanoE, 3000IU SID) for at least 3 weeks. 

Real time PCR tests performed by Cornell University Animal Health Diagnostic Center reported negative results for the flavivirus equine hepacivirus and positive results for equine parvovirus (EqPV-H) with Ct values of 32.36. EqPV-H, a frequent equine infection [34,35], has been associated with and shown to induce hepatitis following inoculation although many EqPV-H infections are also asymptomatic [36,37,38].

**Figure 1:**
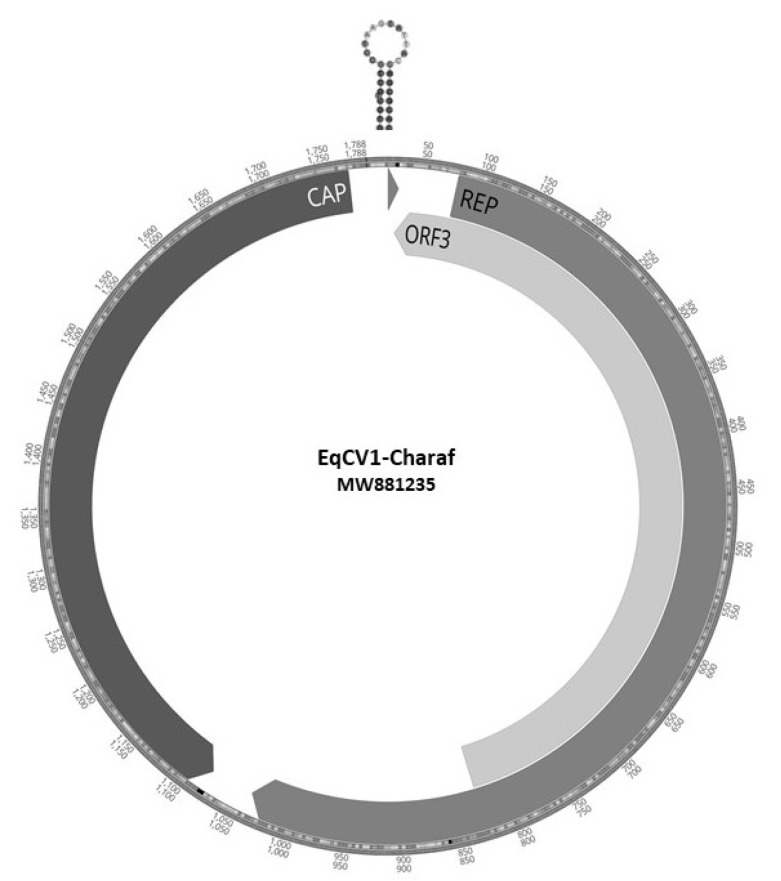
ORF map of EqCV1 showing location and sequence of origin of replication and of three major ORFs.

PCRs followed by amplicons Sanger sequencing were then used to fill two gaps in the genome allowing the complete equine circovirus (EqCV) genome to be assembled. The EqCV1 genome was 1788 bases long, with a GC content of 53%, with three major ORFs encoding replicase (Rep) capsid (Cap) proteins and ORF3 of unknown function (GenBank MW881235) (Figure 1). 

The Rep protein was 327 amino acids long, sharing aa-identities of 74% with elk circovirus (Genbank MN585201) followed by PCV2 (GenBank EU148505.1) with 73% identity. The 231 amino acid long capsid protein was also closest to elk circovirus capsid (63%) (GenBank MN585201.1) followed by PCV1 (GenBank NP_065679.1) with 61.7%, PCV2 (GenBank NP_937957) (60.2%), and bat associated circovirus 2 (GenBank YP_007974238) with 48% identity. A third major ORF of a 274 amino acid protein was also identified located antisense over the rep ORF. Its closest known relative is the ORF3 from PCV2 (GenBank YP_006355434.1) at 54% identity over 38% of protein. This protein found in PCV1 (204 amino acids) and PCV2 (104 amino acids) is believed to trigger apoptotic activity in infected cells [39]. 

The Rep contained all three expected rolling circle replication motifs I (VFTLNN), II (PHLQG) and III (YCGK) [40]. The C-terminal region possessed the ATP-dependent helicase Walker A (GPPGCGKS), Walker B (VLDDY) and Walker C (ITSN) [40] motifs. The origin of replication stem loop had a 14 base pairs stem and a 12 base loop with a nearly canonical (TAGTATTAC) sequence (AAGTATTAC) [40]. 

**Figure 2:**
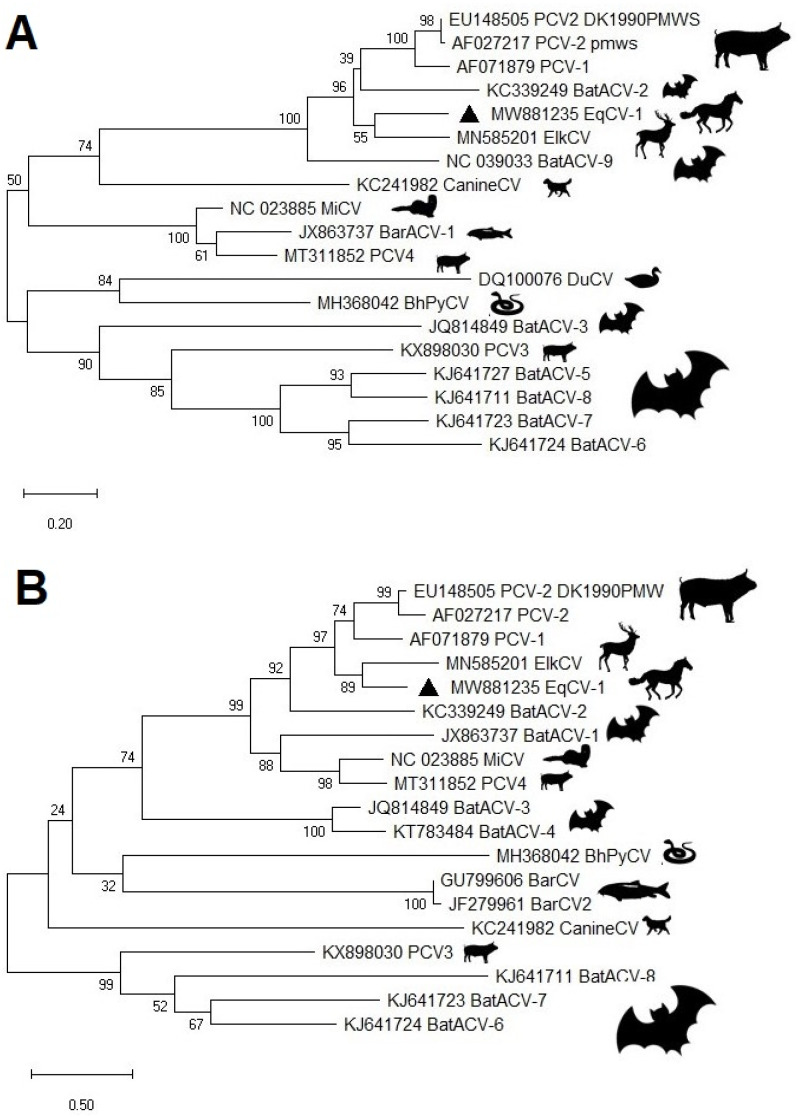
(**A**). Phylogenetic analysis of EqCV1-Charaf replicase. (**B**). Phylogenetic analysis of EqCV1-Charaf capsid. The scale indicates amino acid substitutions per position. The amino acid (aa) pairwise alignments were performed with Genious software using the in-built MAFFT algorithm. The phylogenetic trees were constructed using the Maximum likelihood method with substitution model: Le Gascuel 2008 based model with gamma-distributed (G+) for Rep and Cap in MEGA software version X [33,41].

Phylogenetic analyses were then performed showing that the EqCV1 rep and cap proteins clustered with PCV1/2, as well as elk and bat 2 (insectivorous Rhinolophus ferrumequinum or greater horseshoe bat) circoviruses (Figure 2). 

According to the ICTV, the member of the same circovirus species should share >80% nucleotide identity over their entire genome, and >70% amino acid identity between their Cap proteins [1]. When using the complete genome the closest relative to EqCV1-Charaf was elk circovirus with 74.4% identity over 58% of genome. Its closest capsid protein, also from elk circovirus, was only 63% identical. EqCV1 is therefore proposed as a new species in the genus Circovirus, and the first circovirus species found in a member of the Equus genus.

## 4. Discussion

The equine circovirus genome characterized here is most closely related to a Canadian elk circovirus reported once and to pig circoviruses PCV1 and PCV2 distributed world-wide. Four porcine circovirus species are currently known that are either non-pathogenic (PCV1), pathogenic (PCV2), possibly pathogenic (PCV3) [42,43], or still of unknown pathogenicity (PCV4) [44]. PCV2 has been extensively studied due to its significant impact on porcine health often in the context of co-infections with other viruses [7]. There are numerous reports that pig circoviruses can have a wide host range. PCV2 and/or PCV3 have been detected by PCR in numerous other species [45] including ungulate species such as wild boars [46,47], water buffalos [48], cows [49], and others ungulates [50,51], as well as in raccoon dogs [52], rats [53], and dogs [54]. One study reported viral replication and seroconversion of calves following inoculation with cell grown PCV2 [55]. Serological surveys of bovine, equine, and human sera from Canada and the US did not detect anti-PCV2 antibodies indicating infections with PCV2 to be rare or non-existent in cows, horses, and humans [56,57]. 

The common detection of PCV1 and PCV2 in pig world-wide [58] therefore provides a readily available source of virus from which the divergent EqCV1 may have evolved. Based on the estimated mutation rate of PCV2 the ~30% nucleotide genetic distance between EqCV1 and PCV1, PCV2, or ElkCV indicates that cross species transmission between these ungulates may have occurred as long as centuries ago [59]. PCR detection of PCV2 in non-ungulate species may also reflect cross species transmissions that have not (yet) led to viral adaptation and wide-spread infections in the new hosts. Ungulates may be better hosts for PCV2 and PCV3 resulting in the high rate of detection in wild boars and water buffalos [45,60]. The detection of distinct PCV1/PCV2-related circoviruses in an elk and now a horse may therefore reflect successful adaptation to these hosts starting from progenitors such as PCV1 or PCV2 although a reverse direction of transmission from elks or horses to pigs cannot be excluded. 

Attributing disease causality to a new virus without fulfilling Koch’s postulates is challenging. Typically, this begins with elimination of pathogens known to be able to cause the observed lesion, and is followed by epidemiologic, clinical, pathological and microbiological investigations that test this new virus-disease association. Here a horse with a mild case of hepatitis was infected with a circovirus but also viremic at low levels (real time PCR Cut off 34) with the hepatotropic EqPV-H. Although many asymptomatic EqPV-H infections have been reported [34,35] EqPV-H detection in this horse does provides a conceivable explanation for its hepatitis [36,38]. Whether EqCV1 played a leading, supporting, or no role in this case of hepatitis or has the potential to cause other equine diseases will require further studies.

## Data Availability

All sequencing data and circovirus genomes deposited in GenBank under SRA and accession number SAMN18737161 for the Illumina reads and MW881235 for the EqCV1-Charaf genome.
